# A role for the NPM1/PTPN14/YAP axis in mediating hypoxia-induced chemoresistance to sorafenib in hepatocellular carcinoma

**DOI:** 10.1186/s12935-022-02479-0

**Published:** 2022-02-08

**Authors:** Dengke Zhang, Fazong Wu, Jingjing Song, Miaomiao Meng, Xiaoxi Fan, Chenying Lu, Qiaoyou Weng, Shiji Fang, Liyun Zheng, Bufu Tang, Yang Yang, Jianfei Tu, Min Xu, Zhongwei Zhao, Jiansong Ji

**Affiliations:** Zhejiang Provincial Key Laboratory of Imaging Diagnosis and Minimally Invasive Intervention Research, Lishui Hospital of Zhejiang University, The Fifth Affiliated Hospital of Wenzhou Medical University, Lishui, 323000 China

**Keywords:** PTPN14, Hepatocellular carcinoma, Hypoxia, NPM1, YAP, Nuclear translocation

## Abstract

**Background:**

Tumor microenvironments are characterized by resistance to chemotherapeutic agents and radiotherapy. Hypoxia plays an important role in the development of tumor resistance, as well as the generation of metastatic potential. YAP also participates in the regulation of hypoxia-mediated chemoresistance, and is negatively regulated by protein tyrosine phosphatase non-receptor type 14 (PTPN14).

**Methods:**

The PTPN14 expression in hepatocellular carcinoma (HCC) tissues were evaluated by qRT-PCR, western blot and tissue microarrays. The effect of PTPN14 on HCC progression was investigated in vitro and in vivo.

**Results:**

Here, we report that PTPN14 expression was downregulated in HCC tissues and cell lines. Silencing PTPN14 significantly enhanced proliferation, migration, invasion of HepG2 cells in vitro and tumor growth and metastasis in vivo, whereas overexpression of PTPN14 significantly inhibited these abilities in SK-Hep1 cells. We also found that hypoxia-induced nuclear translocation and accumulation of PTPN14 led to resistance to sorafenib in HCC cells. Further mechanistic studies suggested that NPM1 regulates PTPN14 localization, and that NPM1 regulates YAP by retaining PTPN14 in the nucleus under hypoxic conditions.

**Conclusions:**

These data suggest that a therapeutic strategy against chemoresistant HCC may involve disruption of NPM1-mediated regulation of YAP by retaining PTPN14 in the nucleus under hypoxic conditions.

**Supplementary Information:**

The online version contains supplementary material available at 10.1186/s12935-022-02479-0.

## Background

Hepatocellular carcinoma (HCC) is the fifth most common cancer worldwide, and is the second most common cause of cancer death in the world [1, 2]. Despite substantial progress in cancer therapeutics, prognosis in patients with HCC remains low [3, 4]. Common predisposing conditions for HCC include end-stage liver fibrosis and cirrhosis, as well as chronic inflammation secondary to viral infections, chemical exposure, autoimmune diseases and metabolic conditions [5, 6].

Solid tumors such as HCC are characterized by areas of hypoxia, for various reasons including the absence of access to a blood supply [7, 8]. Tumor hypoxia is associated with radioresistance, chemoresistance and metastasis, eventually leading to cancer progression and poor prognosis [9, 10]. Nuclear translocation and activation of yes-associated protein (YAP) by hypoxia contributes to chemoresistance in hepatocellular carcinoma cells [11, 12]. YAP binds to hypoxia-inducible factor-1α (HIF-1α) and sustains HIF-1α protein stability to promote HCC cell glycolysis under hypoxic stress [11, 13].

Protein tyrosine phosphatase non-receptor type 14 (PTPN14) interacts with and negatively regulates the oncogenic function of YAP [14, 15]. PTPN14 suppresses pancreatic cell proliferation and transformation [16, 17]. PTPN14 knockdown enhances osteosarcoma cells colony formation [8, 18]. Nevertheless, the role of PTPN14 and its relationship with YAP in HCC remains largely unknown.

PTPN14 can be localized in both the cytoplasm and nucleus [19]. In the current study, we show that PTPN14 localizes to the nucleus under hypoxic conditions, even though no classical nuclear localization sequence (NLS) has been reported or predicted in PTPN14. It has been speculated that the nuclear localization of PTPN14 may be achieved by its interaction with partner protein(s) with an NLS sequence. Nucleophosmin (NPM1/B23) interacts with several protein partners and is localized primarily in nucleoli. However, NPM1 shuttles between the nucleus and the cytoplasm, and sustained cytoplasmic distribution contributes to its tumor-promoting activities [20, 21]. NPM1 is a nucleolar phosphoprotein that is involved in many cellular processes and has both oncogenic and growth suppressing activities. NPM1 interacts with activating transcription factor 5 (ATF5) protein and promotes proteasome- and caspase-dependent ATF5 degradation in HCC cells [22, 23]. NPM1 interacts with several protein partners, modulating their stability. Importantly, NPM1 appears to play a fundamental role in nuclear localization of these proteins. According to the BioGRID database, PTPN14 is predicted to interact with NPM1. Since the mechanisms of hypoxia-induced chemoresistance in HCC have not been fully elucidated, we conducted the following study to examine the roles of PTPN14, YAP and NPM1 in generation of resistance to the chemotherapeutic agent sorafenib.

## Methods

### Tissue samples

Quantitative PCR and western blotting were performed on fresh HCC samples that had been collected from patients who underwent surgical resection at Lishui Hospital of Zhejiang University (Zhejiang, China). The Ethics Committee of Lishui Hospital of Zhejiang University approved this study. The protocols were in accordance with the Declaration of Helsinki. Informed consent was obtained from all patients.

### Tissue microarray

HCC tissue microarrays (TMA) were purchased from Servicebio Technology Co. (Wuhan, China), and a standard protocol was used for immunostaining of the TMAs. Immunohistochemistry was performed to determine the PTPN14 (1:150, Santa Cruz Biotechnology, Santa Cruz, CA, USA) levels. Briefly, paraffin was removed from sections, after which they were rehydrated and placed in a microwave oven for heat-induced epitope retrieval. Endogenous peroxide activity was quenched with 3% H_2_O_2_. Sections were then blocked in 5% fetal bovine serum (FBS) and incubated overnight at 4 °C with primary antibodies. The following day, horseradish peroxidase secondary antibodies (Invitrogen) were applied and sections were exposed to 3 3′-diaminobenzidine (Zhongshan Golden Bridge Biotechnology, Beijing, China) and counterstained with hematoxylin.

The histochemistry score (H-score) was calculated as previously described [24]. H-Scores were calculated as follows: H-score = (percentage of cells of weak intensity × 1) + (percentage of cells of moderate intensity × 2) + (percentage of cells of strong intensity × 3). Samples with an H-score of more than the median were considered to be high; those less than the median were considered low.

### Cell culture

We obtained human HCC cells (Huh7, HepG2 and SK-HEP1) and LO2 cells from the Shanghai Institute of Cell Biology, Chinese Academy of Sciences (Shanghai, China). We cultured HepG2 and LO2 cells in RPMI 1640 medium (Gibco BRL, Rockville, MD, USA) with 10% FBS (Gibco). Huh7 cells were cultured in DMEM medium (Gibco) with 10% FBS. SK-HEP1 cells were grown in MEM medium (Gibco) with 10% FBS. All cell lines were maintained in medium supplemented with 1% penicillin/streptomycin (Gibco) at 37 °C, in a humidified atmosphere of 5% CO_2_. Hypoxia was induced by incubating cells at 37 °C in an atmosphere containing 93% N_2_, 5% CO_2_ and 2% O_2_ for 24 h. Atmospheric conditions were maintained using a triple-gas incubator(Huaxi Electronic Tec., China). Triple-gas incubator by controlling the input of O_2_ or N_2_, with zirconia (ZrO_2_) sensor to achieve control over O_2_ content, to perform three gas control of O_2_, N_2_ and CO_2_. When O_2_ gas concentration was less than 19%, the advanced N_2_ gas was used, after reaching the O2 concentration set value, and then the way CO_2_ gas was re fed, to ensure the accuracy of CO_2_ gas concentration and O_2_ concentration. When O_2_ gas concentration was more than 23%, the method of using advanced O_2_ gas, after reaching the O_2_ concentration set value, and then the way CO_2_ gas was re fed, to ensure the accuracy of CO_2_ gas concentration and O_2_ concentration.

### Cell transfection and stable cell line generation

For PTPN14 knockdown, shRNAs for human *PTPN14* were designed and constructed in a GV112 lentiviral expression plasmid (Shanghai Genechem Co., Ltd., Shanghai, China). shRNA plasmids were constructed using *PTPN14* target (5′-GGTCTACAGCAACAAACTTGT-3′´) and a scrambled sequence (5′-TTTAGACTTTATGAGCTAA-3′) was used as the negative control.

For PTPN14 overexpression, using cDNA from the human PTPN14 gene, we amplified a fragment encoding the full-length PTPN14 open reading frame sequence using PCR and then cloned it into GV341 lentiviral expression plasmids (Shanghai Genechem Co., Ltd.). Lentivirus with an empty vector served as the negative control.

For production of recombinant lentiviruses, we co-transfected HEK293T cells with their respective recombinant expression lentivectors together with enveloped, packaged plasmids using the Lipofectamine™ 2000 transfection reagent (Life Technologies, Gaithersberg, MD, USA). The viral supernatants were harvested 48 h after transfection, and viral titers were measured. The recombinant lentivirus was infected into HCC cells for 48 h in medium containing 6 μg/ml polybrene (Sigma-Aldrich Co., St. Louis, MO, USA). Fresh culture medium containing 2 μg/ml puromycin was added to select stable transfected cell lines.

siRNAs targeting NPM1 (siNPM1) and YAP (siYAP) were synthesized by Genepharm Technologies (Shanghai, China). Full-length NPM1 was amplified by PCR and subcloned into the pcDNA3-based expression vector (Invitrogen, Carlsbad, CA, USA). Plasmids and all siRNA transfections were performed using Lipofectamine 2000. For transient transfection, we transfected cells with plasmids or siRNAs at various concentrations as indicated for 48 h before performing functional assays.

### Total RNA extraction and quantitative real-time polymerase chain reaction (qRT-PCR)

Total RNA was extracted using TRIzol (Life Technologies). We performed reverse transcription into cDNA using the SuperScript III cDNA synthesis kit (Life Technologies). We employed cDNA samples (2 μl) for qRT-PCR using SYBR Green PCR Master Mix (Takara, Dalian, China) for 40 cycles on an ABI Prism 7500 detection system (Life Technologies). The following primers were used for qRT-PCR: PTPN14, forward, 5ʹ-TCCCTGTAAAGGACAATCAT-3ʹ and reverse, 5ʹ-GTGGCAAACAACCGAGAA-3ʹ; GAPDH, forward, 5ʹ-TCAAGAAGGTGGTGAAGCAGG-3ʹ and reverse, 5ʹ-TCAAAGGTGGAGGAGTGGGT-3ʹ. We used the 2^−ΔΔCt^ method for relative quantification of gene expression and normalized the data to β-actin expression.

### Western blotting

Proteins was extracted using RIPA lysis buffer (Beyotime Institute of Biotechnology, Jiangsu, China) according to the manufacturer’s instructions. For nuclear proteins, we used NE-PER™ Nuclear and Cytoplasmic Extraction Reagents (Thermo Fisher Scientific, Rockford, IL, USA) according to the manufacturer’s instructions. We determined protein concentrations using the bicinchoninic acid method (BCA, Pierce, Rockford, IL, USA). Then, 30 µg of total protein was separated using 10% sodium dodecyl sulfate polyacrylamide gel electrophoresis (SDS-PAGE), followed by electrotransfer onto PVDF membranes (Millipore, Bedford, MA, USA). Following blocking, we incubated membranes with antibodies specific for PTPN14 (Santa Cruz Biotechnology, Santa Cruz, CA, USA), YAP or phosphorylated-YAP (p-YAP) (Ser127) (Cell Signaling Technology, Danvers, MA, USA), GAPDH or lamin B (Abcam, Cambridge, MA, USA). Finally, we incubated blots with goat anti-rabbit or anti-mouse secondary antibodies (Santa Cruz Biotechnology) and visualized signals with enhanced chemiluminescence (Pierce).

### Cell viability assay

We seeded cells in 100 µl growth medium at 8 × 10^3^ cells per well in 96-well plates. Following overnight incubation, we treated cells with or without sorafenib (Sigma-Aldrich), then incubated the cells with 10 μl of MTT (5 mg/ml, Cat.11465007001, Sigma-Aldrich) for 4 h at 37 °C. We measured cell viability using a microplate reader (BioTek, Winooski, VT, USA) at 570 nm. IC_50_ was calculated using GraphPad Prism 5. Each experiment was performed at least three times using separate cultures.

### Colony formation assay

We seeded cells into 6-well plates at about 500 cells/well and cultured them in RPMI-1640 containing 10% fetal bovine serum. At day 14, the plates were fixed in 4% paraformaldehyde and stained with 1% crystal violet sequentially. We counted colonies with  ≥ 50 cells manually under a dissection microscope.

### Invasion and migration assays

After treatment, cells were placed in Transwell chambers (8 μm pore; BD Biosciences, San Jose, CA, USA) as previously described. For the invasion assay, 1 × 10^5^ cells were seeded into the upper chamber coated with Matrigel in a cell invasion system (BD Biosciences). The lower chamber was filled with medium. After incubation, invading cells were fixed with methanol, visualized by staining with 0.1% crystal violet and counted. For the migration assay, 5 × 10^4^ cells were seeded into the upper chamber and the lower chamber was filled with medium. After incubation, the cells that had migrated through the membrane were fixed, stained and counted.

### Immunofluorescent staining and confocal microscopy

Confocal microscopic imaging of immunofluorescent staining. Briefly, cells were washed and applied to slides by cytospinning. Then, the cells were fixed for 20 min in PBS containing 4% paraformaldehyde, permeabilized in 1% Triton X-100 for 20 min, then incubated in blocking buffer (5% FBS in PBS) for 30 min. The cells were rinsed in PBS and incubated overnight at 4 °C in dilution buffer containing primary antibodies against PTPN14 (1:100, Santa Cruz Biotechnology, Santa Cruz, CA, USA) or YAP (1:200, Cell Signaling Technology, Danvers, MA, USA). The cells were washed three times with PBS before being incubated with an appropriate fluorochrome-conjugated secondary antibody (Alexa Fluor 488- or Alexa Fluor 594-conjugated secondary antibody, Invitrogen) for 1 h at 37 °C in the dark. After nuclear counterstaining with 4, 6-diamidino-2-phenylindole (DAPI, Beyotime, China), coverslips were fixed with mounting medium and cells were visualized using a light microscope (Nikon, Tokyo, Japan).

### Co-immunoprecipitation

For co-immunoprecipitation (Co-IP), cells subjected to hypoxia for various durations were harvested. Total lysates were incubated with the human PTPN14 antibody (Santa Cruz Biotechnology) overnight. After overnight incubation, we added protein-G or protein-A sepharose CL-4B (Invitrogen) and incubated the mixture for another 4 h. We then subjected the suspension to several pull-down assays, washed the mixture five times in PBS, and PTPN14-associated protein complexes were separated using SDS-PAGE. Finally, we immunoblotted the membranes using antibodies against human NPM1 or PTPN14.

### In vivo tumorigenicity and pulmonary metastasis

Animal studies were approved by the Lishui Hospital of Zhejiang University, Zhejiang, China. Male athymic BALB/c nude mice (4–5 weeks old) were used for this study (Shanghai Slac Laboratory Animal Co., China). For in vivo tumorigenicity experiments, we subcutaneously injected equal numbers (1 × 10^7^) of transduced HCC cells into each mouse flank. We measured tumor volumes using the formula: volume (mm^3^) = (width)^2^ × length/2, and mice were euthanized using CO_2_ inhalation (CO_2_ flow rate, 20% of chamber volume per minute) 5 weeks after injection.

To generate in vivo models of pulmonary metastasis, we injected luciferase transduced HCC cells into nude mice through the tail vein. After about 8 weeks, mice were injected with D-luciferin (150 mg/kg), anesthetized with 5% isofluorane, and tumor metastases were visualized and imaged using a whole-body fluorescent imaging system. At the end of the experiments, mice were euthanized using CO_2_ inhalation (CO_2_ flow rate, 20% of chamber volume per minute), and the whole lungs were excised.

### Statistical analysis

Data were expressed as mean  ±  SD. The differences in mean values between two groups were analyzed using the Student’s *t* test, and differences in mean values between several groups were analyzed using one-way ANOVA. All statistical analyses were performed using GraphPad Prism 5.0 software. *p*  < 0.05 was considered statistically significant.

## Results

### Downregulation of PTPN14 in HCC tissues

**Fig. 1:**
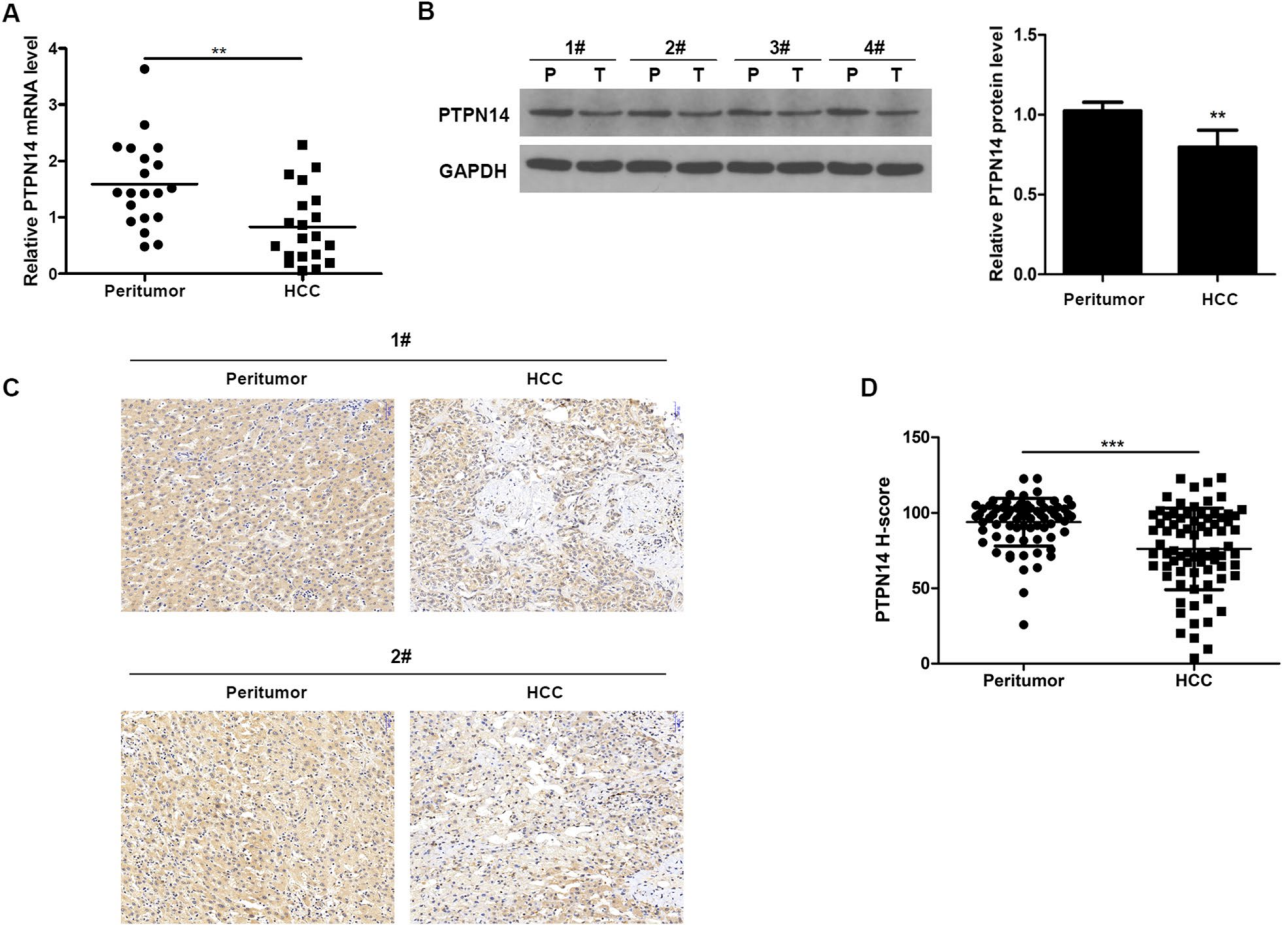
Decreased expression of PTPN14 in HCC. **A** PTPN14 mRNA levels in 20 paired HCC and corresponding peritumoral liver tissues were evaluated by qRT-PCR. **B** PTPN14 protein levels in tumor tissues (T) and the paired adjacent non-tumor tissues (P) from four HCC patients were examined by western blotting. **C** Representative cases of PTPN14 IHC staining in HCC and corresponding peritumoral liver tissues by tissue microarray. Scale bar = 50 μm. **D** Relative H-score of PTPN14 in paired HCC and corresponding peritumoral liver tissue samples (*n* = 77). Statistical significance was determined by Student’s *t *tests. **p < 0.01, ***p < 0.001

We measured PTPN14 mRNA levels in 20 paired HCC and corresponding peritumoral liver tissues using qRT-PCR. We found significantly lower levels of PTPN14 expression in HCC tissues than in peritumor tissue (p  < 0.01) (Fig. 1A). Consistent with this result, western blots demonstrated that PTPN14 protein was present in higher levels in peritumor tissues than in HCC (Fig. 1B). This result was confirmed by immunohistochemical staining for PTPN14 in sections of tissue taken from patients with HCC (Fig. 1C, D).

### PTPN14 suppresses HCC growth and tumorigenesis in vitro and in vivo

**Fig. 2:**
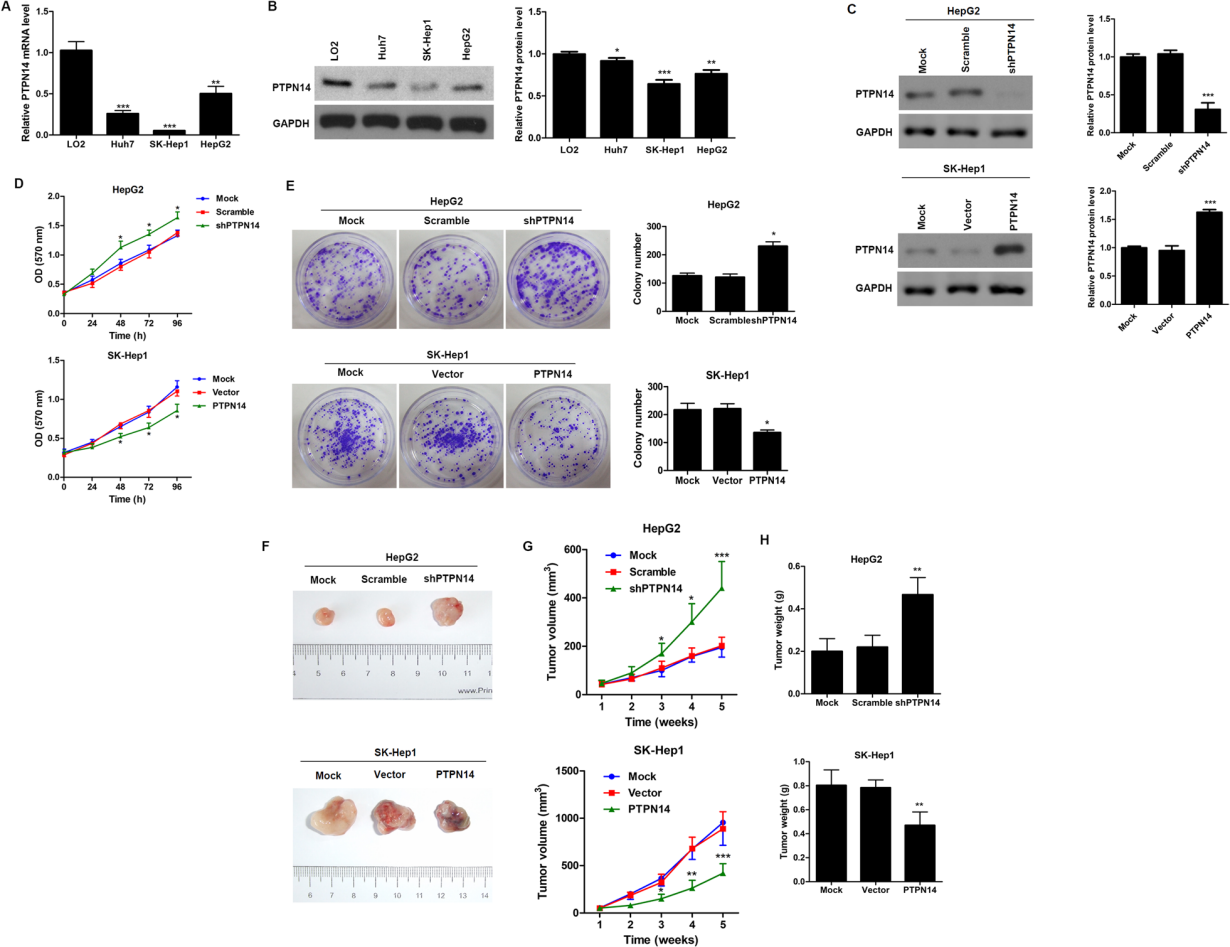
PTPN14 inhibits HCC growth and tumorigenesis in vitro and in vivo. The expression of PTPN14 in different HCC cell lines with varied metastatic potential was analyzed by qRT-PCR (**A**) and western blotting (**B**). **C** Verification of the efficiency of PTPN14 knockdown in HepG2 cells or overexpression in SK-Hep1 cells. **D** Cell proliferation was determined by MTT assays in HepG2 cells with stable PTPN14 knockdown and SK-Hep1 cells with PTPN14 stable overexpression at the indicated times. **E** Representative images and quantification of cell clones in HepG2 cells with stable PTPN14 knockdown and SK-Hep1 cells with PTPN14 stable overexpression as determined by a colony formation assay. HepG2 cells with stable PTPN14 knockdown and SK-Hep1 cells with PTPN14 stable overexpression were injected subcutaneously into nude mice (1 × 10^7^ cells per mouse, five mice per group). Mice and tumors were evaluated 5 weeks post-injection and representative images are shown (**F**). Tumor growth curve during the 5-week study period (**G**) and tumor weights (**H**) are shown. Data are shown as the mean  ±  SEM. Statistically significant differences were determined by Student’s *t *tests. *p  < 0.05; **p  < 0.01 compared with the respective controls

We next examined PTPN14 expression in various HCC cell lines (HepG2, Huh7 and SK-Hep1) using qRT-PCR (Fig. 2A) and western blotting (Fig. 2B), and compared expression in these cells to that of a normal hepatic cell line (LO2). We found that PTPN14 expression was significantly lower in HCC lines than in the normal hepatic cell line. To test the effect of PTPN14 on cell proliferation in vitro, we stably knocked down PTPN14 expression in HepG2 cells and overexpressed PTPN14 in SK-Hep1 cells (Fig. 2C). We found that PTPN14 knockdown significantly enhanced proliferation, while overexpression significantly inhibited proliferation over a 96-h time course (Fig. 2D, E).

To confirm these results in vivo, we subcutaneously injected HepG2 cells with stable knockdown of PTPN14 as well as SK-Hep1 cells with stable overexpression of PTPN14 into nude mice and measured tumor growth over 5 weeks (Fig. 2F). The tumor volume was significantly higher in HepG2 cells with PTPN14 knockdown and was significantly lower in SK-Hep1 cells with PTPN14 overexpression (Fig. 2G). Similarly, tumor weight was significantly higher in HepG2 cells with PTPN14 knockdown and significantly lower in SK-Hep1 cells with PTPN14 overexpression (Fig. 2H). These results suggest that, in vivo, PTPN14 suppresses tumor growth and that, conversely, absence of PTPN14 promotes tumor growth in HCC.

### PTPN14 suppresses HCC invasion and migration in vitro and in vivo

**Fig. 3:**
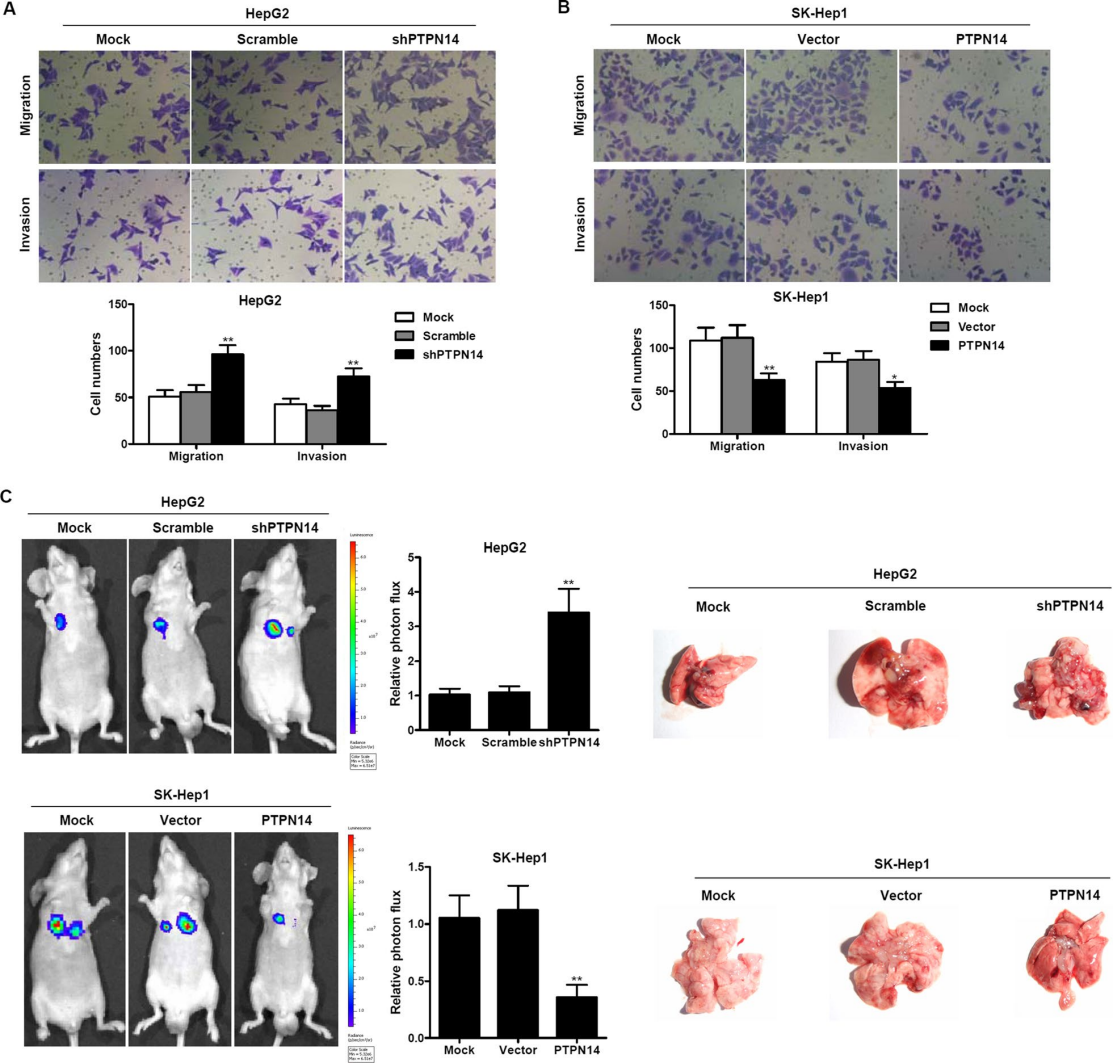
PTPN14 suppresses HCC invasion and migration in vitro and in vivo. The migration and invasion of HepG2 cells with stable PTPN14 knockdown (**A**) and SK-Hep1 cells with PTPN14 stable overexpression (**B**) was assessed by a Transwell assay. The number of migrating and invading cells was counted in at least three different fields per sample. The data are shown as the means  ±  SD (*n*  = 3). *p  < 0.05; **p < 0.01 compared with the respective controls. **C** The pulmonary metastasis of HepG2 cells with stable PTPN14 knockdown and SK-Hep1 cells with PTPN14 stable overexpression was monitored by bioluminescence imaging in vivo. Pulmonary metastasis was observed using a whole-body fluorescent imaging system. Right, gross view of lung metastatic nodules. Relative photon flux of the two groups was calculated. The data are shown as the mean  ±  SD (*n*  = 5). **p  < 0.01 compared with the respective controls

We next examined the effects of PTPN14 on HCC cell invasion and migration in vitro (Fig. 3A, B). Transwell assays revealed that when PTPN14 was stably knocked down, migration and invasion were significantly greater than in the controls (Fig. 3A). Conversely, when PTPN14 was stably overexpressed, migration and invasion were significantly less than in the controls (Fig. 3B). We then measured the effect of PTPN14 knockdown or overexpression on the extent of pulmonary metastasis. We injected transduced HCC cells into nude mice via the tail vein. After about 8 weeks, the mice were anesthetized. We imaged the mice using whole-body fluorescent imaging (Fig. 3C). We found that PTPN14 knockdown significantly enhanced pulmonary metastases, whereas overexpression significantly inhibited metastases. These results suggest that PTPN14 expression inhibits tumor cell migration and invasion in HCC cells, and that conversely, PTPN14 blockade promotes tumor invasion and migration.

### Nuclear translocation of PTPN14 is induced under hypoxic conditions and is essential for hypoxia-induced drug resistance

**Fig. 4:**
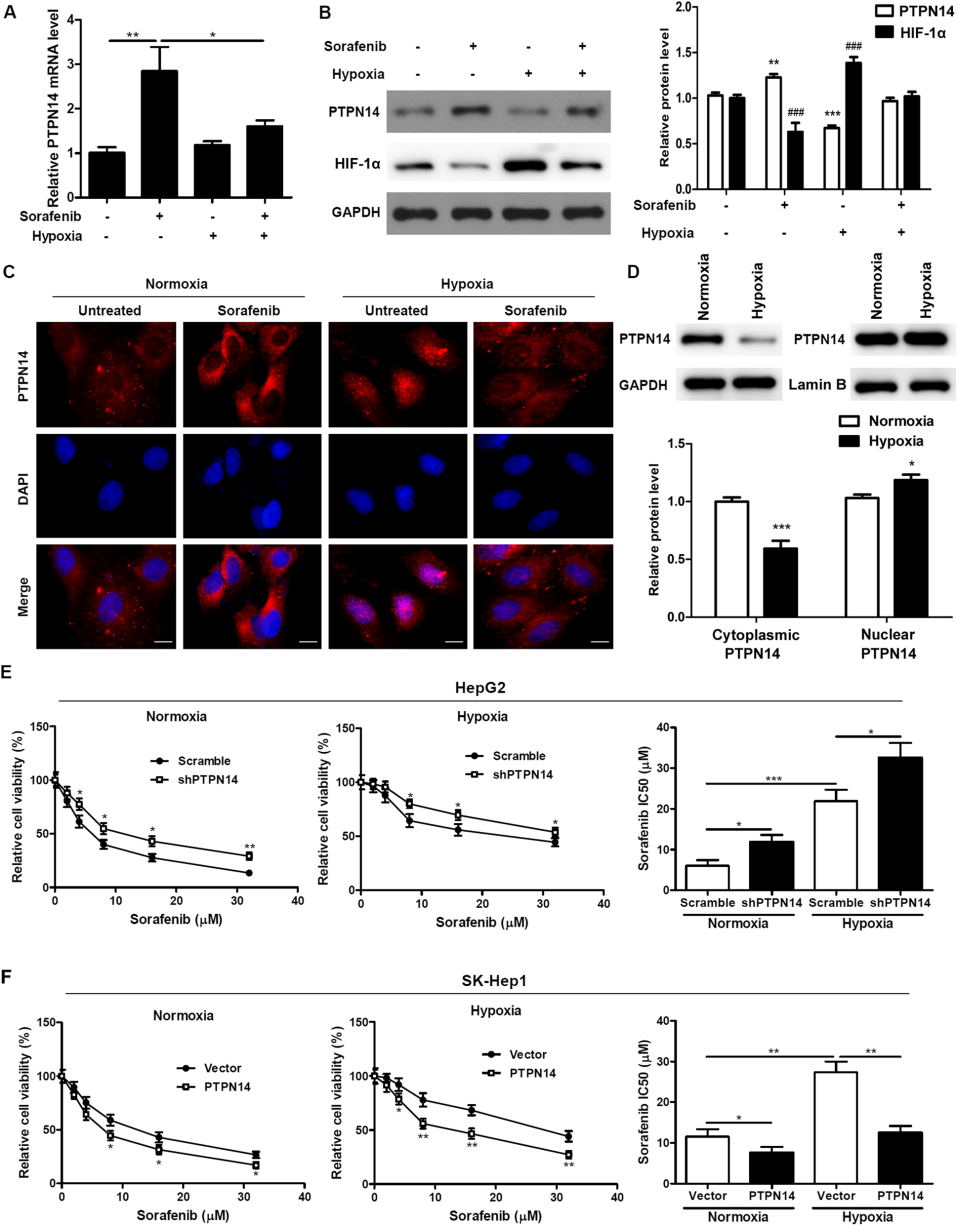
Nuclear translocation of PTPN14 is induced under hypoxia and is required for hypoxia-induced drug resistance. PTPN14 mRNA levels (**A**) and protein levels (**B**) in HepG2 cells upon sorafenib (5 µM) treatment under hypoxia (2% O_2_) for 24 h. **C** Representative immunofluorescence staining of PTPN14 in HepG2 cells upon sorafenib (5 µM) treatment under normoxia (20% O_2_) or hypoxia (2% O_2_) for 24 h. Scale bar = 20 μm. **D** PTPN14 protein levels within the cytoplasm and nucleus under normoxic and hypoxic conditions.E andF. HepG2 cells with stable PTPN14 knockdown (**E**) and SK-Hep1 cells with PTPN14 stable overexpression (**F**) were treated with the indicated doses of sorafenib under normoxia or hypoxia for 72 h. Cell viability was calculated by MTT assays. The IC_50_ values derived from the dose–response curves are presented

To study the effects of PTPN14 in mediating the effects of hypoxia-induced drug resistance, we first examined the effects of the tyrosine kinase inhibitor sorafenib (5 µM) on PTPN14 expression in HepG2 cells under both normoxic (20% O_2_) and hypoxic (2% O_2_) conditions for 24 h. We found that PTPN14 expression was significantly elevated by sorafenib treatment under normoxic conditions at both the mRNA level (Fig. 4A) and the protein level (Fig. 4B); this elevation persisted, though at lower levels, under hypoxic conditions. These findings suggest that sorafenib causes elevation of PTPN14 expression, a circumstance that may lead to inhibition of cancer cell proliferation, migration and invasion. We confirmed these findings using confocal microscopy. We examined the subcellular localization of PTPN14 in response to sorafenib under normoxic and hypoxic conditions, and found that sorafenib induced cytoplasmic PTPN14 expression, and hypoxia induced nuclear translocation of PTPN14; however, sorafenib-induced PTPN14 cytoplasmic translocation was reduced under hypoxic conditions (Fig. 4C). Moreover, PTPN14 protein levels in the cytosol were significantly reduced under hypoxic conditions as determined by WB (Fig. 4D).

We then looked at the effects of sorafenib and hypoxia over 72 h in HCC cells where PTPN14 was either knocked down or overexpressed. In HepG2 cells, PTPN14 knockdown elevated the IC_50_ of sorafenib, both in normoxic and hypoxic conditions (Fig. 4E), although hypoxia decreased the sensitivity of HepG2 cells to sorafenib, as demonstrated by an elevated IC_50_. Conversely, in SK-Hep1 cells, PTPN14 overexpression decreased the IC_50_ of sorafenib, both under normoxic and hypoxic conditions (Fig. 4F), despite the fact that the cells were less sensitive to sorafenib under hypoxic conditions. Taken together, these results suggest that hypoxia both increases PTPN14 nuclear translocation and decreases cell sensitivity to the chemotherapeutic agent sorafenib.

### NPM1 regulates PTPN14 localization under hypoxic conditions

**Fig. 5:**
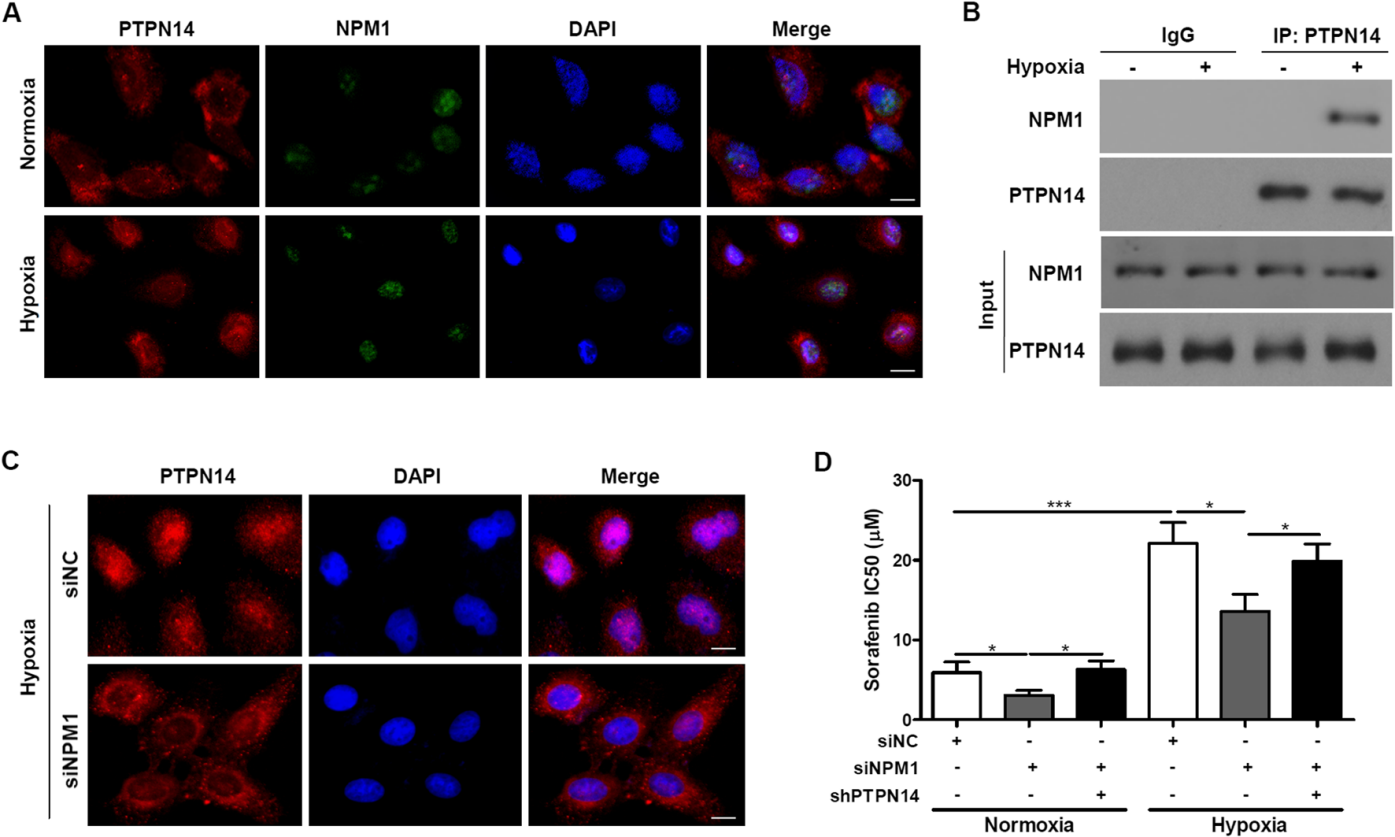
NPM1 regulates PTPN14 localization under hypoxia. **A** Representative immunofluorescence staining of the localization of PTPN14 and NPM1 in HepG2 cells under normoxia (20% O_2_) or hypoxia (2% O_2_) for 24 h. Scale bar = 20 μm. **B** Total lysate was harvested from HepG2 cells after hypoxia for 0 or 24 h. After the cells had been immunoprecipitated using PTPN14 antibody, their interaction with NPM1 was examined using the indicated antibodies. **C** Representative immunofluorescence staining of PTPN14 in HepG2 cells transfected with NPM1 siRNA (siNPM1) under hypoxic conditions (2% O_2_) for 24 h. Scale bar = 20 μm. **D** The IC_50_ was measured using an MTT assay after 72 h of sorafenib treatment

Using three web-based NLS prediction programs, PSORT, cNLS mapper and NLSdb, we found that PTPN14 does not contain any classical NLS sequences. Thus, we hypothesized that nuclear localization of PTPN14 is achieved by its interaction with other known nuclear proteins. Because the predicted interaction between PTPN14 and the protein nucleophosmin (NPM1/B23) are reported in BioGRID, we performed colocalization staining for PTPN14 and NPM1 in HepG2 cells, both under normoxic and hypoxic conditions for 24 h. We found that PTPN14 and NPM1 colocalized in the nucleus under hypoxic conditions, and that hypoxia triggered the release of NPM1 from nucleoli (Fig. 5A). To verify this result, we performed a co-precipitation experiment. We added anti-PTPN14 antibodies to precipitated proteins harvested from HepG2 cells treated with or without hypoxia (Fig. 5B). We found that NPM1 co-precipitated with PTPN14 after treatment with hypoxia but not without hypoxia. This result suggests that hypoxic stress triggers nuclear localization of PTPN14.

We also performed localization assays of PTPN14 in HepG2 cells with NPM1 knockdown under normoxic and hypoxic conditions for 24 h. We found that PTPN14 was mainly distributed in the cytoplasm under hypoxic conditions in NPM1-knockdown cells, whereas it remained in the nucleus of cells with normal expression of NPM1 (Fig. 5C). Taken together, these results suggest that NPM1 interacts with PTPN14 and that this interaction is required for nuclear retention of PTPN14 under hypoxic conditions.

Next, we examined the influence of PTPN14 or NPM1 knockdown on the effect of sorafenib on cell viability. The IC_50_ was measured using an MTT assay after 72 h of sorafenib treatment. We found that under normoxic conditions, knockdown of NPM1 increased sorafenib sensitivity of HepG2 cells, whereas this effect was abolished by PTPN14 knockdown (Fig. 5D). These findings indicate that NPM1 regulates PTPN14 localization under hypoxic conditions and mediates sorafenib resistance through PTPN14.

### PTPN14 enhances sensitivity to sorafenib via repression of YAP under hypoxic conditions

**Fig. 6:**
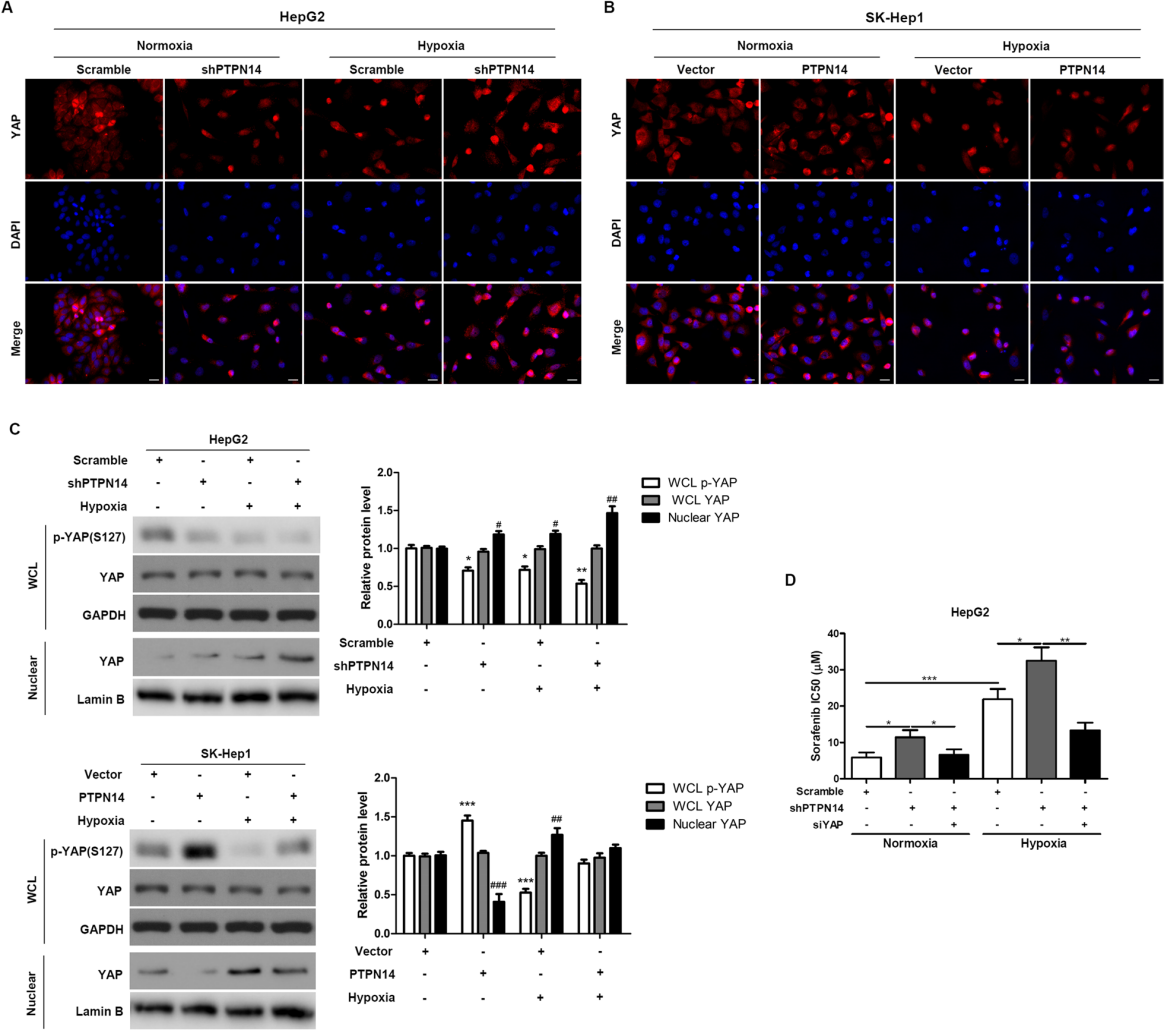
PTPN14 regulates sensitivity to sorafenib through YAP under hypoxia. Representative immunofluorescence staining of the localization of YAP in HepG2 cells with stable PTPN14 knockdown (**A**) and SK-Hep1 cells with PTPN14 stable overexpression (**B**) under normoxia (20% O_2_) or hypoxia (2% O_2_) for 24 h. Scale bar = 20 μm. **C** The expression of p-YAP and total YAP in the whole cell lysate (WCL) and nuclear fractions in HepG2 cells with stable PTPN14 knockdown and SK-Hep1 cells with PTPN14 stable overexpression under hypoxic conditions (2% O_2_) for 24 h was examined by western blotting. **D** The IC_50_ was measured using an MTT assay after 72 h of sorafenib treatment. *p < 0.05; **p < 0.01; ***p < 0.001

We next sought to determine whether PTPN14 activity inactivated YAP, and exerted an anti-tumor effect. To explore the role of YAP in the mechanism of action of PTPN14 with respect to sorafenib sensitivity, we performed localization experiments in HepG2 cells with and without stable PTPN14 knockdown, under both normoxic and hypoxic conditions. We found that YAP translocated to the nucleus under hypoxic conditions for 24 h, and knockdown of PTPN14 enhanced hypoxia-induced nuclear translocation of YAP (Fig. 6A). In SK-Hep1 cells overexpressing PTPN14, hypoxia-induced nuclear translocation of YAP was attenuated by PTPN14 overexpression (Fig. 6B).

We then studied the effects of PTPN14 knockdown and overexpression on the levels of YAP and phosphorylated YAP (p-YAP) under normoxic and hypoxic conditions for 24 h. In HepG2 cells with stable knockdown of PTPN14, nuclear translocation of YAP induced by hypoxia was promoted by PTPN14 knockdown. The levels of p-YAP were diminished by PTPN14 knockdown under hypoxic conditions (Fig. 6C). Conversely, in SK-Hep1 cells that were stably overexpressing PTPN14, nuclear translocation of YAP was significantly diminished compared to control cells under both normoxic and hypoxic conditions (Fig. 6C).

We also measured the IC_50_ for sorafenib using an MTT assay after 72 h of treatment of the cells with either PTPN14 or YAP knockdown (Fig. 6D). We found that hypoxia significantly increased the IC_50_ of sorafenib, and that this effect was further augmented by PTPN14 knockdown. However, the effect of hypoxia and PTPN14 knockdown was abolished by knockdown of YAP expression. Taken together, these data suggest that under hypoxic conditions, PTPN14 enhances sensitivity to sorafenib via repression of YAP.

### NPM1 regulates YAP by retaining PTPN14 in the nucleus under hypoxic conditions

**Fig. 7:**
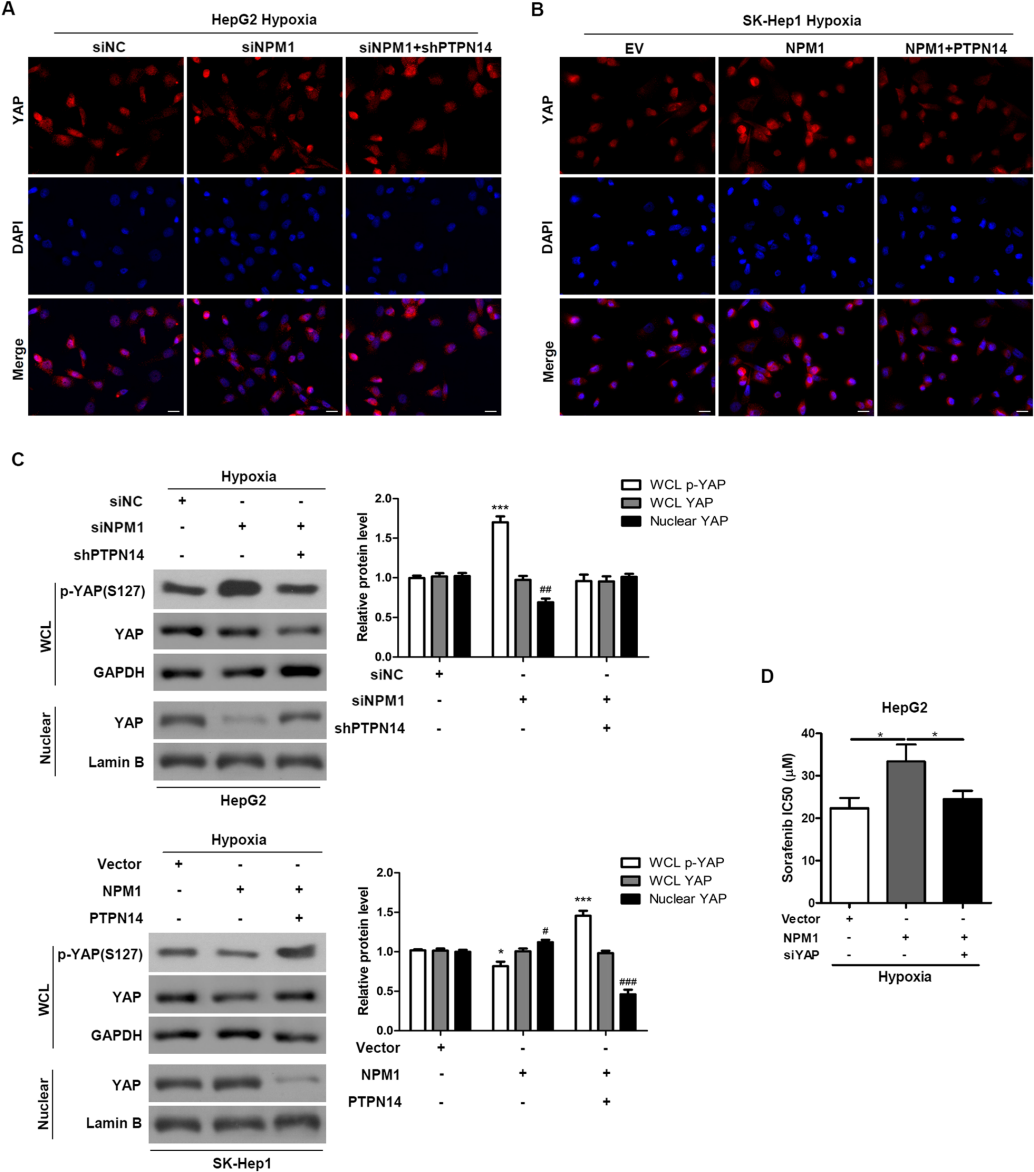
NPM1 regulates YAP by retaining PTPN14 in the nucleus under hypoxia. Representative immunofluorescence staining of the localization of YAP in HepG2 cells with NPM1 or/and PTPN14 knockdown (**A**) and SK-Hep1 cells with NPM1 or/and PTPN14 overexpression (**B**) under hypoxia (2% O_2_) for 24 h. Scale bar = 20 μm. **C** The expression of p-YAP and total YAP in the whole cell lysate (WCL) and nuclear fractions of HepG2 cells with NPM1 or/and PTPN14 knockdown and SK-Hep1 cells with NPM1 or/and PTPN14 overexpression under hypoxia (2% O_2_) for 24 h was examined by western blotting. **D** The IC_50_ was measured using an MTT assay after 72 h of sorafenib treatment. *p < 0.05

To further explore the mechanism of action of PTPN14 under hypoxic conditions, we studied the effects of NPM1 under hypoxic conditions (2% O_2_). We found that NPM1 knockdown prevented translocation of YAP to the nucleus, an effect that was partially reversed by PTPN14 knockdown (Fig. 7A, C). Conversely, NPM1 overexpression enhanced nuclear translocation of YAP in a manner that was partially reversed by PTPN14 overexpression (Fig. 7B, C). Furthermore, under hypoxic conditions, levels of p-YAP were significantly increased by NPM1 knockdown, in a manner that was partially reversed by PTPN14 knockdown (Fig. 7C). Levels of p-YAP were significantly enhanced by overexpression of NPM1 and PTPN14 together, but not by overexpression of NPM1 alone. When both NPM1 and PTPN14 were overexpressed, nuclear translocation of YAP was significantly inhibited.

**Fig. 8:**
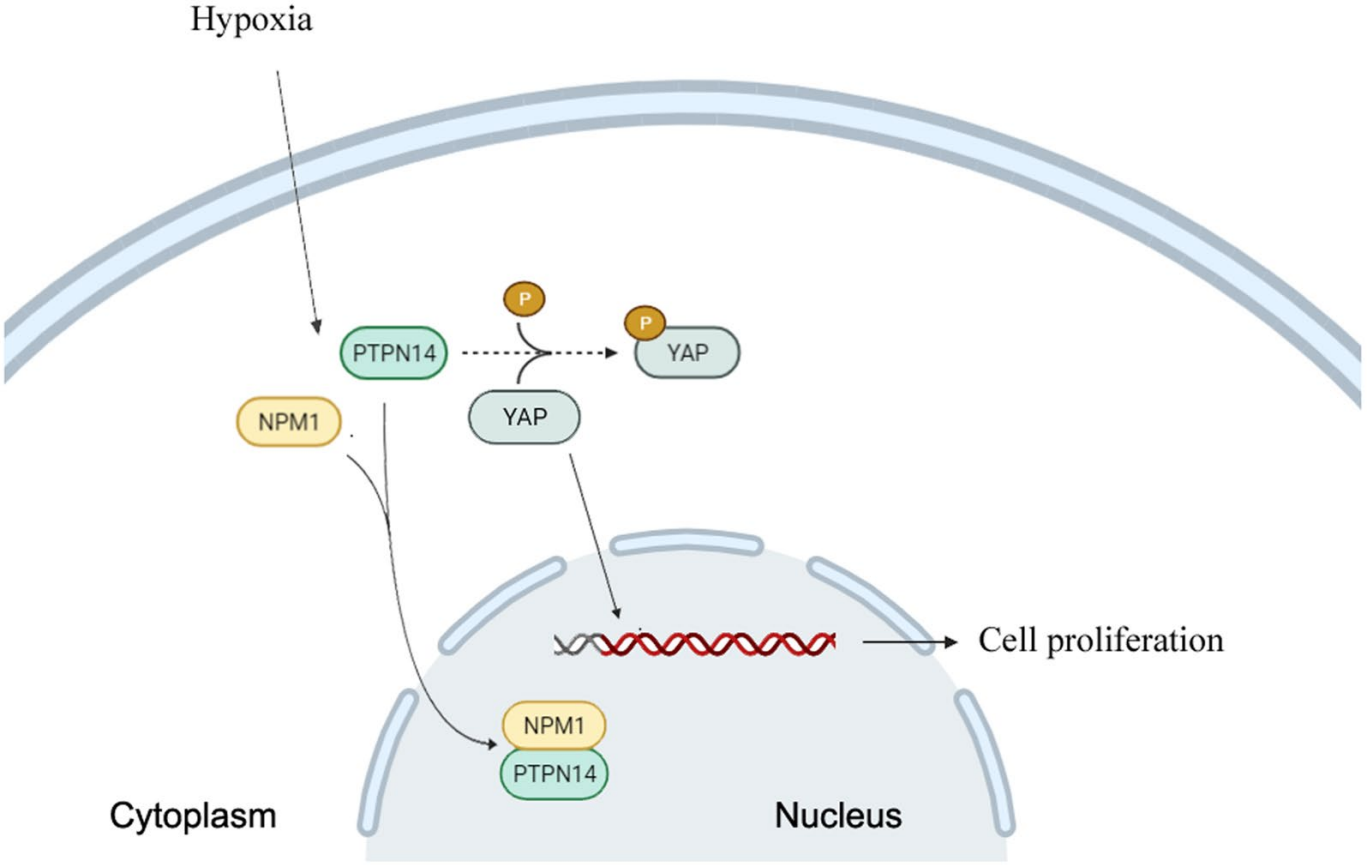
Schematic diagram of hypoxia-induced NPM1 combined with PTPN14 attenuating inhibition of YAP

Finally, we found that NPM1 overexpression increased the IC_50_ of sorafenib in a manner that was partially reversed by knockdown of YAP expression (Fig. 7D). Taken together, these results suggest that NPM1 is responsible, at least in part, for regulation of YAP by retaining PTPN14 in the nucleus under hypoxic conditions (Fig. 8). Furthermore, inhibition of PTPN14 causes HCC to become more chemoresistant to sorafenib under hypoxic conditions [11] (Additional file 1).

## Discussion

The findings of this study elucidated certain aspects of the mechanism by which hypoxia induces chemoresistance to sorafenib in HCC. Our data suggested that the mechanism may be initiated by hypoxic stress, triggering the translocation of NPM1 from the nucleolus into the nucleus. We speculate that once in the nucleus, NPM1 complexes with PTPN14, preventing its interaction with YAP. Increased YAP activity promotes cell proliferation, survival, stemness, migration, invasion and chemoresistance.

PTPN14 is a nonreceptor protein tyrosine phosphatase that is involved in regulating a variety of cellular processes, including cell adhesion, cell growth, differentiation and cancer progression. PTPN14 mutations have been identified in a variety of human malignancies, including breast cancer, colon cancer, and skin basal cell carcinoma [25–27], and thus PTPN14 has been implicated as a putative tumor and metastasis suppressor. Several potential substrates for PTPN14, including β-catenin [28], p130Cas [29], RIN1 and PRKCD [30], and YAP [14], are all related to tumor progression and metastasis.

Liu et al. [14] demonstrated that YAP was a direct substrate of PTPN14. The authors showed that inhibition of YAP’s transcriptional co-activator function by PTPN14 was mediated by direct protein interactions, the result of which was increased levels of cytoplasmic phosphorylated YAP, which is inactive. They further showed that knockdown of PTPN14 induced nuclear retention of YAP and increased YAP-dependent cell migration. Mello et al. [16], studying pancreatic cancer cells, found that PTPN14 suppressed the YAP activity that was necessary for p53 tumor suppressor function; a p53 transcriptional activation mutant showed elevated tumor suppression capacity because of hyperactivation of PTPN14.

Dai et al. [11] reported that YAP participates in hypoxia-induced chemoresistance. They found that activation of YAP under hypoxic conditions correlated with resistance to the chemotherapeutic agent SN38, a topoisomerase I inhibitor. This mechanism appeared to be independent of HIF1-α [13]. These findings suggested that YAP was involved in more than one pathway responsible for tumorigenesis, increasing the list of possible anti-tumor targets.

The trigger for all of these hypoxia-related events may involve NPM1. Yang et al. [20] used a model of oxidation-mediated nucleolar stress and found that cellular stresses triggered the glutathionylation of NPM1, causing it to dissociate from nucleic acids and thereby become active. In their model, activated NPM1 was essential for the stress-induced activation of p53. Several lines of evidence suggest that NPM1 activation is a common pathway for responses to several kinds of cellular stress, including exposure to chemotherapeutic agents [31, 32], heat shock [33, 34] and UV radiation [35–37]. Li et al. [38] reported that NPM1 is induced by hypoxia and protects human breast cancer cells against hypoxic cell death. Consistent with previous studies, the present study demonstrated that hypoxia triggered the release of NPM1 from nucleoli, suggesting that NPM1 expression is hypoxia responsive.

In HCC, Liu et al. [14] found that NPM1 was highly expressed and facilitated cell proliferation, whereas ATF5 inhibited proliferation. Their findings demonstrated a mechanistic link between elevated NPM1 expression and depressed ATF5 in HCC, suggesting that regulation of ATF5 by NPM1 participates in cell proliferation in HCC.

The mechanisms by which NPM1 modulates the effects of cell cycle pathways have been the subject of several studies, including that of Di Matteo et al. [39], who studied the interaction of NPM1 with the tumor suppressor Fbw7γ. They found that NPM1 bound to a nucleolar localization signal (NoLS) in Fbw7γ. The authors suggested that the NoLS interaction may serve as a potential target in cancer therapeutics.

The mechanism suggested in our present study involves a variation on the tumor-suppressive mechanisms mentioned in these previous studies. In our model, NPM1 binds to PTPN14 under hypoxic conditions, retaining PTPN14 in the nucleus and preventing its interaction with YAP, that under normal conditions results in YAP maintaining its inactive (phosphorylated) state. As a result, HCC continues to proliferate and metastasize, resulting in larger hypoxic tumor volumes and continuing the cycle of hypoxic stress.

## Conclusions

Our mechanistic study suggested that NPM1 mediates PTPN14 localization, and regulates YAP by retaining PTPN14 in the nucleus under hypoxic conditions in HCC. Taken together, our data suggest that a therapeutic strategy against hypoxia-induced sorafenib-resistance to HCC may involve disruption of the NPM1/PTPN14/YAP axis.

## Supplementary Information

**Additional file 1: Figure S1.** HE detection of lung metastasis sections.

## Data Availability

The datasets used and/or analyzed during the current study are available from the corresponding author upon reasonable request.

## Abbreviations

HCC: Hepatocellular carcinoma
YAP: Yes-associated protein
HIF-1α: Hypoxia-inducible factor-1α
PTPN14: Protein tyrosine phosphatase non-receptor type 14
NLS: Nuclear localization sequence
NPM1/B23: Nucleophosmin
ATF5: Activating transcription factor 5
TMA: Tissue microarrays
FBS: Fetal bovine serum
H-score: Histochemistry score
qRT-PCR: Quantitative real-time polymerase chain reaction
Co-IP: Co-immunoprecipitation
