# Supplementary figures and images for: A role for the NPM1/PTPN14/YAP axis in mediating hypoxia-induced chemoresistance to sorafenib in hepatocellular carcinoma
Source: Cancer Cell Int. 2022 Feb 8;22:65. doi: 10.1186/s12935-022-02479-0 (PMC8822852; doi:10.1186/s12935-022-02479-0)

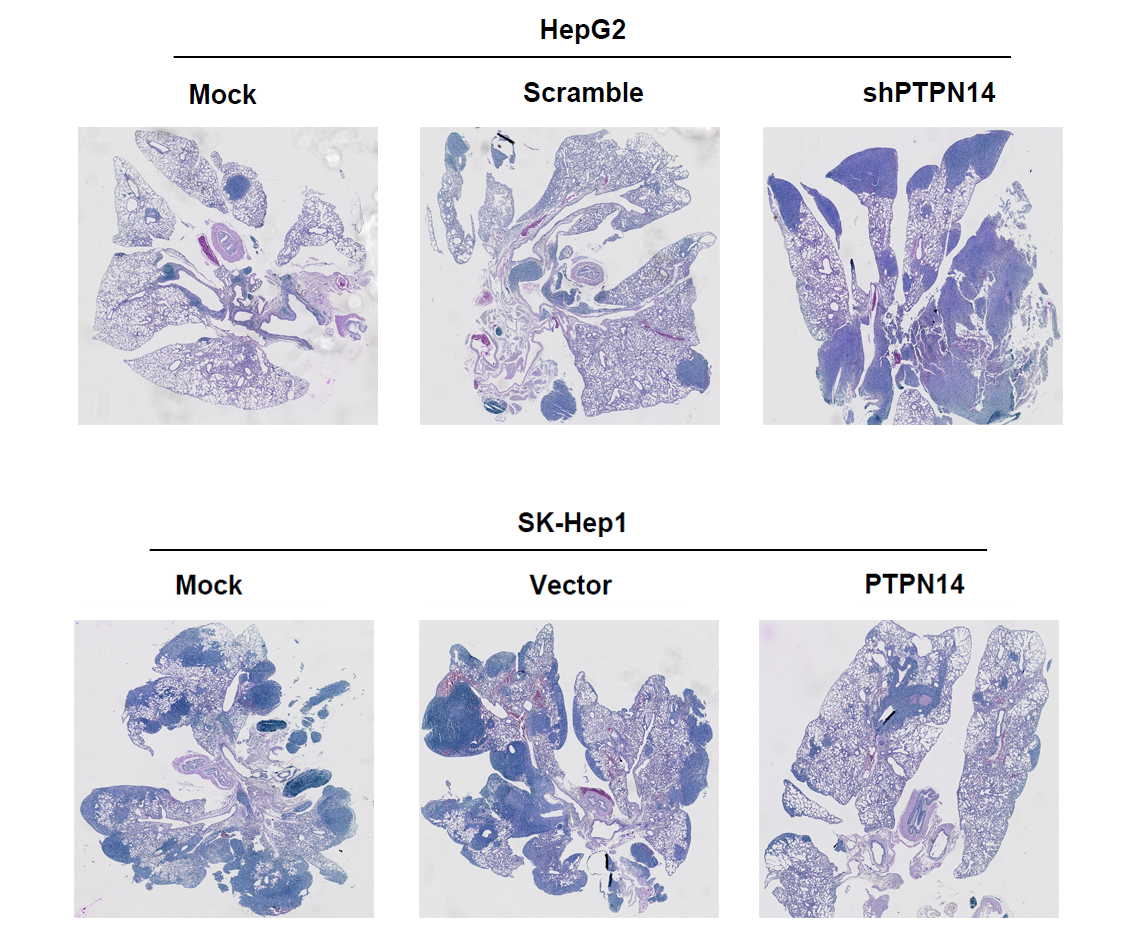

Supplement: Supplementary file 1 — Additional file 1: Figure S1. HE detection of lung metastasis sections. [file 12935_2022_2479_MOESM1_ESM.tif]
